# A topological approach to synaptic connectivity and spatial memory

**DOI:** 10.1186/1471-2202-16-S1-P44

**Published:** 2015-12-18

**Authors:** Russell Milton, Andrey Babichev, Yuri A Dabaghian

**Affiliations:** 1 Department of Neurobiology and Anatomy, University of Texas Health Science Center at Houston, TX 77030, USA; 2 Neurology-Pediatrics Department, Baylor College of Medicine, Houston, TX 77030, USA; 3 Computational and Applied Mathematics, Rice University, Houston, TX 77005, USA

## 

In the hippocampus, a network of place cells generates a cognitive map of space, in which each cell is responsive to a particular area of the environment -- its place field. The peak response of each cell and the size of each place field have considerable variability. Experimental evidence suggests that place cells encode a topological map of space that serves as a basis of spatial memory and spatial awareness. Using a computational model based on Persistent Homology Theory we demonstrate that if the parameters of the place cells spiking activity fall inside of the physiological range, the network correctly encodes the topological features of the environment. We next introduce parameters of synaptic connectivity into the model and demonstrate that failures in synapses that detect coincident neuronal activity lead to spatial learning deficiencies similar to the ones that are observed in rodent models of neurodegenerative diseases. Moreover, we show that these learning deficiencies may be mitigated by increasing the number of active cells and/or by increasing their firing rate, suggesting the existence of a compensatory mechanism inherent to the cognitive map.
